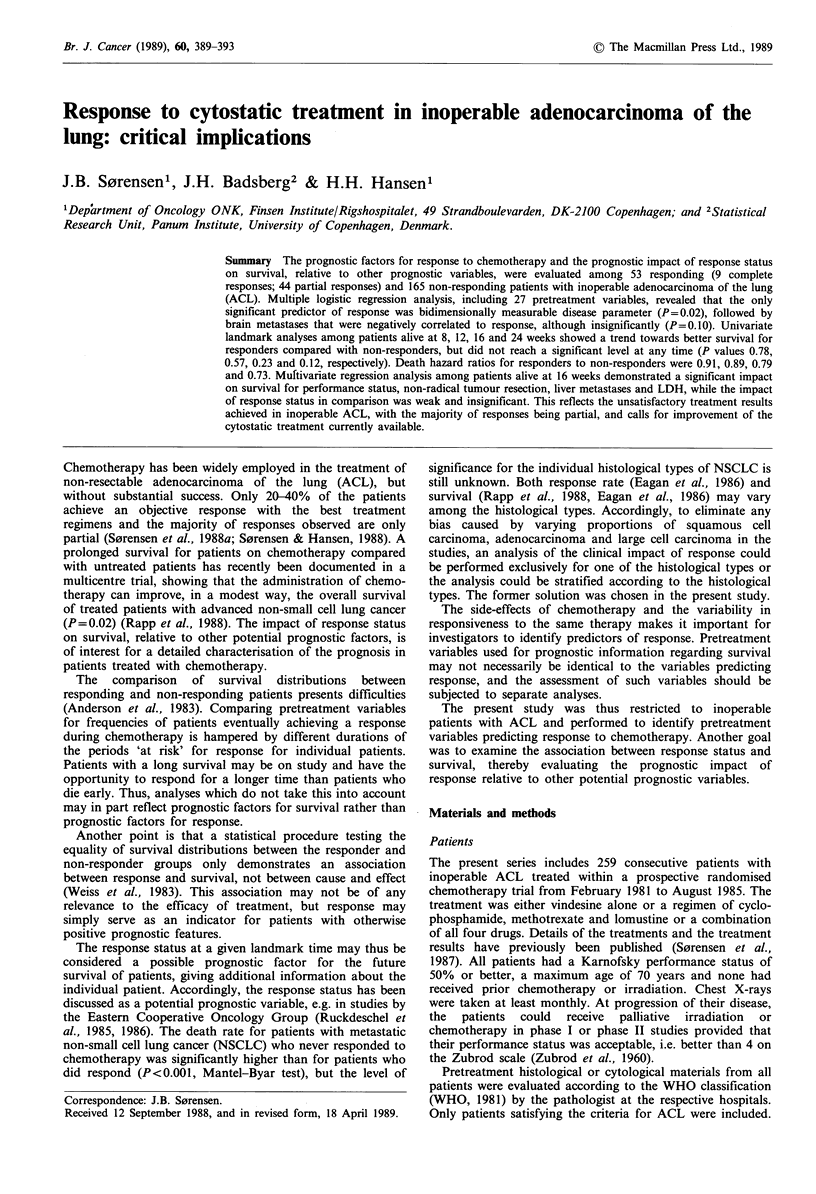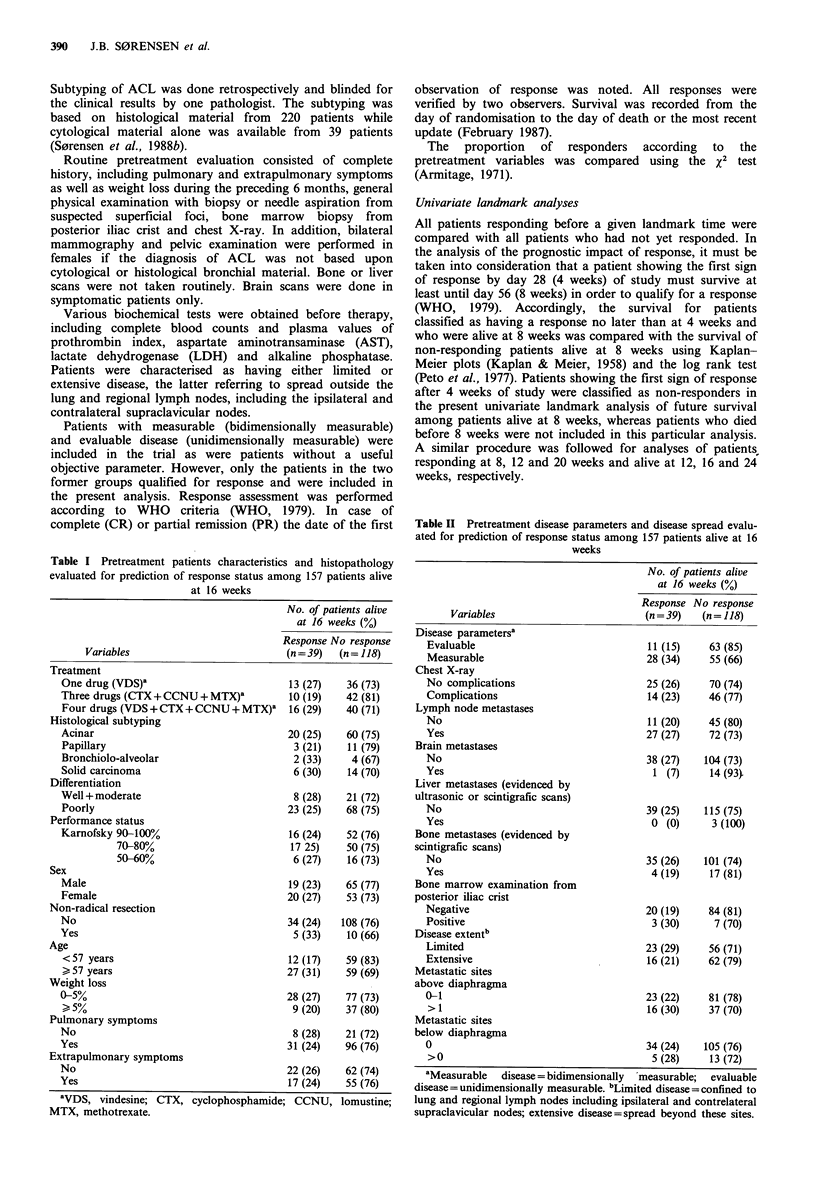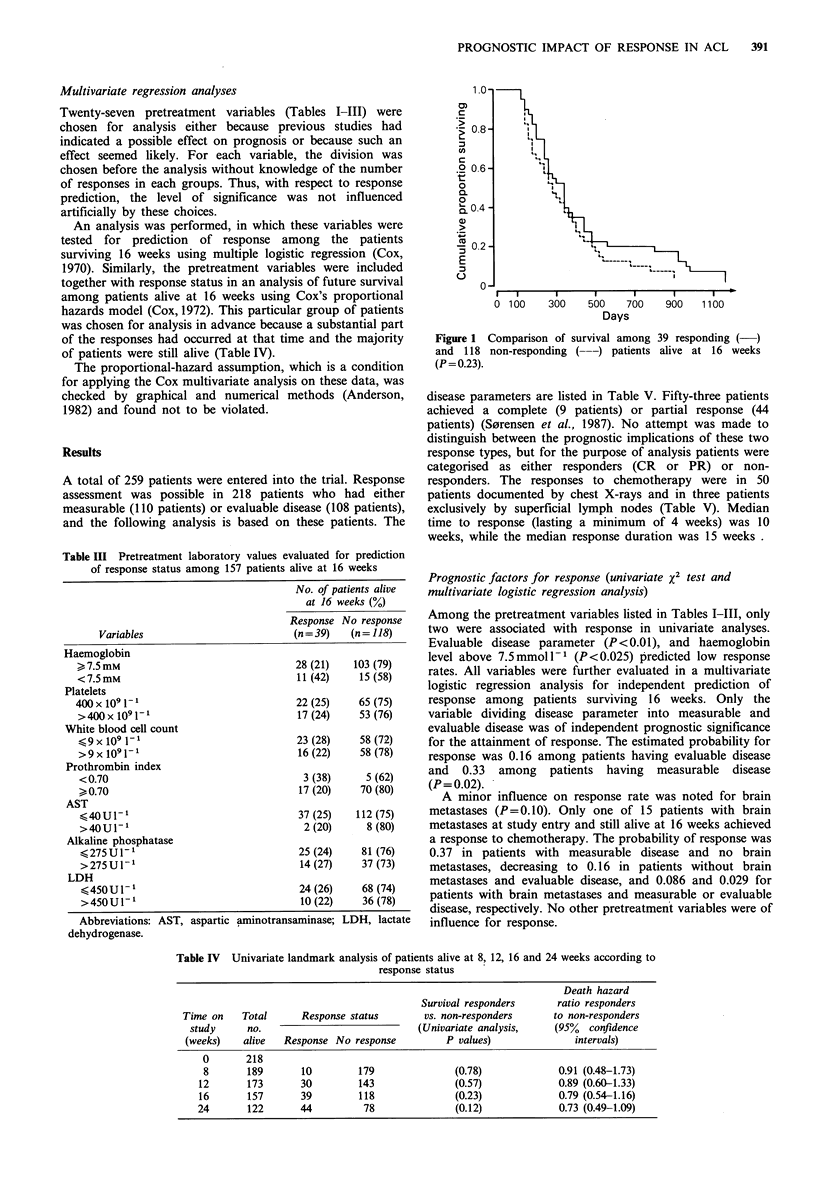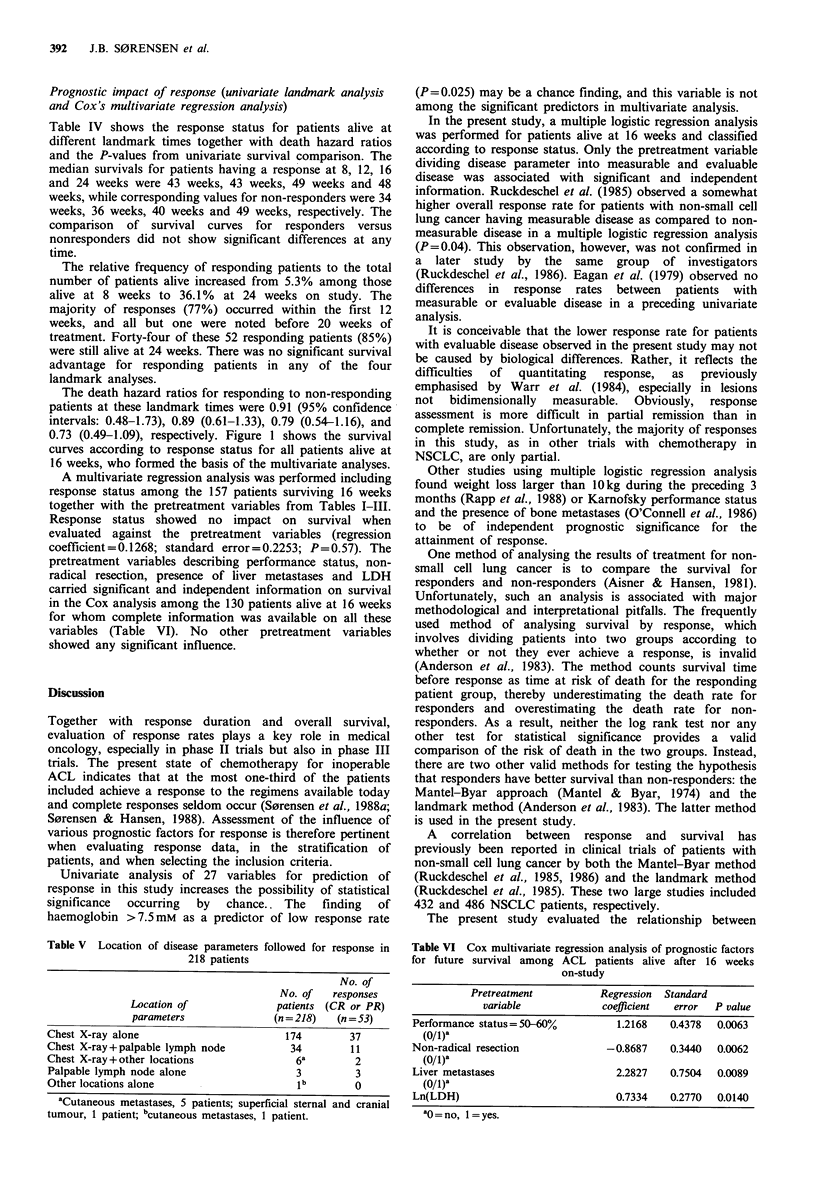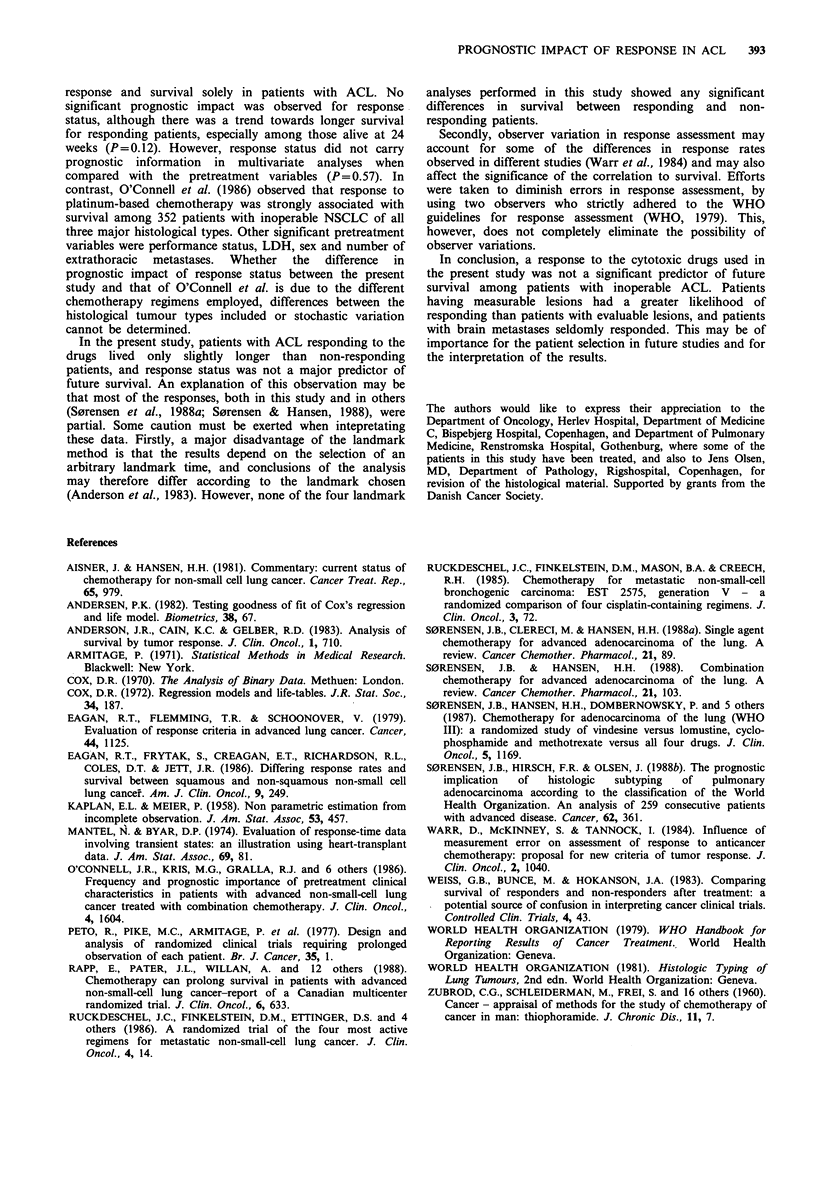# Response to cytostatic treatment in inoperable adenocarcinoma of the lung: critical implications.

**DOI:** 10.1038/bjc.1989.291

**Published:** 1989-09

**Authors:** J. B. SÃ¸rensen, J. H. Badsberg, H. H. Hansen

**Affiliations:** Department of Oncology ONK, Finsen Institute/Rigshospitalet, Copenhagen, Denmark.

## Abstract

The prognostic factors for response to chemotherapy and the prognostic impact of response status on survival, relative to other prognostic variables, were evaluated among 53 responding (9 complete responses; 44 partial responses) and 165 non-responding patients with inoperable adenocarcinoma of the lung (ACL). Multiple logistic regression analysis, including 27 pretreatment variables, revealed that the only significant predictor of response was bidimensionally measurable disease parameter (P = 0.02), followed by brain metastases that were negatively correlated to response, although insignificantly (P = 0.10). Univariate landmark analyses among patients alive at 8, 12, 16 and 24 weeks showed a trend towards better survival for responders compared with non-responders, but did not reach a significant level at any time (P values 0.78, 0.57, 0.23 and 0.12, respectively). Death hazard ratios for responders to non-responders were 0.91, 0.89, 0.79 and 0.73. Multivariate regression analysis among patients alive at 16 weeks demonstrated a significant impact on survival for performance status, non-radical tumour resection, liver metastases and LDH, while the impact of response status in comparison was weak and insignificant. This reflects the unsatisfactory treatment results achieved in inoperable ACL, with the majority of responses being partial, and calls for improvement of the cytostatic treatment currently available.


Br. J. Cancer (1989), 60, 389-393 © The Macmillan Press Ltd., 1989

### Response to cytostatic treatment in inoperable adenocarcinoma of the lung: critical implications

J.B. Sørensen[1], J.H. Badsberg[2] & H.H. Hansen[1]

[1]*Department of Oncology ONK, Finsen Institute/Rigshospitalet, 49 Strandboulevarden, DK-2100 Copenhagen; and* [2]*Statistical Research Unit, Panum Institute, University of Copenhagen, Denmark.*

**Summary** The prognostic factors for response to chemotherapy and the prognostic impact of response status on survival, relative to other prognostic variables, were evaluated among 53 responding (9 complete responses; 44 partial responses) and 165 non-responding patients with inoperable adenocarcinoma of the lung (ACL). Multiple logistic regression analysis, including 27 pretreatment variables, revealed that the only significant predictor of response was bidimensionally measurable disease parameter ($P=0.02$), followed by brain metastases that were negatively correlated to response, although insignificantly ($P=0.10$). Univariate landmark analyses among patients alive at 8, 12, 16 and 24 weeks showed a trend towards better survival for responders compared with non-responders, but did not reach a significant level at any time ($P$ values 0.78, 0.57, 0.23 and 0.12, respectively). Death hazard ratios for responders to non-responders were 0.91, 0.89, 0.79 and 0.73. Multivariate regression analysis among patients alive at 16 weeks demonstrated a significant impact on survival for performance status, non-radical tumour resection, liver metastases and LDH, while the impact of response status in comparison was weak and insignificant. This reflects the unsatisfactory treatment results achieved in inoperable ACL, with the majority of responses being partial, and calls for improvement of the cytostatic treatment currently available.

Chemotherapy has been widely employed in the treatment of non-resectable adenocarcinoma of the lung (ACL), but without substantial success. Only 20–40% of the patients achieve an objective response with the best treatment regimens and the majority of responses observed are only partial (Sørensen *et al.*, 1988*a*; Sørensen & Hansen, 1988). A prolonged survival for patients on chemotherapy compared with untreated patients has recently been documented in a multicentre trial, showing that the administration of chemotherapy can improve, in a modest way, the overall survival of treated patients with advanced non-small cell lung cancer ($P=0.02$) (Rapp *et al.*, 1988). The impact of response status on survival, relative to other potential prognostic factors, is of interest for a detailed characterisation of the prognosis in patients treated with chemotherapy.

The comparison of survival distributions between responding and non-responding patients presents difficulties (Anderson *et al.*, 1983). Comparing pretreatment variables for frequencies of patients eventually achieving a response during chemotherapy is hampered by different durations of the periods 'at risk' for response for individual patients. Patients with a long survival may be on study and have the opportunity to respond for a longer time than patients who die early. Thus, analyses which do not take this into account may in part reflect prognostic factors for survival rather than prognostic factors for response.

Another point is that a statistical procedure testing the equality of survival distributions between the responder and non-responder groups only demonstrates an association between response and survival, not between cause and effect (Weiss *et al.*, 1983). This association may not be of any relevance to the efficacy of treatment, but response may simply serve as an indicator for patients with otherwise positive prognostic features.

The response status at a given landmark time may thus be considered a possible prognostic factor for the future survival of patients, giving additional information about the individual patient. Accordingly, the response status has been discussed as a potential prognostic variable, e.g. in studies by the Eastern Cooperative Oncology Group (Ruckdeschel *et al.*, 1985, 1986). The death rate for patients with metastatic non-small cell lung cancer (NSCLC) who never responded to chemotherapy was significantly higher than for patients who did respond ($P<0.001$, Mantel–Byar test), but the level of significance for the individual histological types of NSCLC is still unknown. Both response rate (Eagan *et al.*, 1986) and survival (Rapp *et al.*, 1988, Eagan *et al.*, 1986) may vary among the histological types. Accordingly, to eliminate any bias caused by varying proportions of squamous cell carcinoma, adenocarcinoma and large cell carcinoma in the studies, an analysis of the clinical impact of response could be performed exclusively for one of the histological types or the analysis could be stratified according to the histological types. The former solution was chosen in the present study.

The side-effects of chemotherapy and the variability in responsiveness to the same therapy makes it important for investigators to identify predictors of response. Pretreatment variables used for prognostic information regarding survival may not necessarily be identical to the variables predicting response, and the assessment of such variables should be subjected to separate analyses.

The present study was thus restricted to inoperable patients with ACL and performed to identify pretreatment variables predicting response to chemotherapy. Another goal was to examine the association between response status and survival, thereby evaluating the prognostic impact of response relative to other potential prognostic variables.

### Materials and methods

### *Patients*

The present series includes 259 consecutive patients with inoperable ACL treated within a prospective randomised chemotherapy trial from February 1981 to August 1985. The treatment was either vindesine alone or a regimen of cyclophosphamide, methotrexate and lomustine or a combination of all four drugs. Details of the treatments and the treatment results have previously been published (Sørensen *et al.*, 1987). All patients had a Karnofsky performance status of 50% or better, a maximum age of 70 years and none had received prior chemotherapy or irradiation. Chest X-rays were taken at least monthly. At progression of their disease, the patients could receive palliative irradiation or chemotherapy in phase I or phase II studies provided that their performance status was acceptable, i.e. better than 4 on the Zubrod scale (Zubrod *et al.*, 1960).

Pretreatment histological or cytological materials from all patients were evaluated according to the WHO classification (WHO, 1981) by the pathologist at the respective hospitals. Only patients satisfying the criteria for ACL were included.

Correspondence: J.B. Sørensen.
Received 12 September 1988, and in revised form, 18 April 1989.

Subtyping of ACL was done retrospectively and blinded for the clinical results by one pathologist. The subtyping was based on histological material from 220 patients while cytological material alone was available from 39 patients (Sørensen et al., 1988b).

Routine pretreatment evaluation consisted of complete history, including pulmonary and extrapulmonary symptoms as well as weight loss during the preceding 6 months, general physical examination with biopsy or needle aspiration from suspected superficial foci, bone marrow biopsy from posterior iliac crist and chest X-ray. In addition, bilateral mammography and pelvic examination were performed in females if the diagnosis of ACL was not based upon cytological or histological bronchial material. Bone or liver scans were not taken routinely. Brain scans were done in symptomatic patients only.

Various biochemical tests were obtained before therapy, including complete blood counts and plasma values of prothrombin index, aspartate aminotransaminase (AST), lactate dehydrogenase (LDH) and alkaline phosphatase. Patients were characterised as having either limited or extensive disease, the latter referring to spread outside the lung and regional lymph nodes, including the ipsilateral and contralateral supraclavicular nodes.

Patients with measurable (bidimensionally measurable) and evaluable disease (unidimensionally measurable) were included in the trial as were patients without a useful objective parameter. However, only the patients in the two former groups qualified for response and were included in the present analysis. Response assessment was performed according to WHO criteria (WHO, 1979). In case of complete (CR) or partial remission (PR) the date of the first observation of response was noted. All responses were verified by two observers. Survival was recorded from the day of randomisation to the day of death or the most recent update (February 1987).

The proportion of responders according to the pretreatment variables was compared using the $\chi^2$ test (Armitage, 1971).

*Univariate landmark analyses*

All patients responding before a given landmark time were compared with all patients who had not yet responded. In the analysis of the prognostic impact of response, it must be taken into consideration that a patient showing the first sign of response by day 28 (4 weeks) of study must survive at least until day 56 (8 weeks) in order to qualify for a response (WHO, 1979). Accordingly, the survival for patients classified as having a response no later than at 4 weeks and who were alive at 8 weeks was compared with the survival of non-responding patients alive at 8 weeks using Kaplan–Meier plots (Kaplan & Meier, 1958) and the log rank test (Peto et al., 1977). Patients showing the first sign of response after 4 weeks of study were classified as non-responders in the present univariate landmark analysis of future survival among patients alive at 8 weeks, whereas patients who died before 8 weeks were not included in this particular analysis. A similar procedure was followed for analyses of patients responding at 8, 12 and 20 weeks and alive at 12, 16 and 24 weeks, respectively.

**Table I** Pretreatment patients characteristics and histopathology evaluated for prediction of response status among 157 patients alive at 16 weeks

| Variables | No. of patients alive at 16 weeks (%) Response (n=39) | No response (n=118) |
|---|---|---|
| Treatment | | |
| One drug (VDS)[a] | 13 (27) | 36 (73) |
| Three drugs (CTX+CCNU+MTX)[a] | 10 (19) | 42 (81) |
| Four drugs (VDS+CTX+CCNU+MTX)[a] | 16 (29) | 40 (71) |
| Histological subtyping | | |
| Acinar | 20 (25) | 60 (75) |
| Papillary | 3 (21) | 11 (79) |
| Bronchiolo-alveolar | 2 (33) | 4 (67) |
| Solid carcinoma | 6 (30) | 14 (70) |
| Differentiation | | |
| Well+moderate | 8 (28) | 21 (72) |
| Poorly | 23 (25) | 68 (75) |
| Performance status | | |
| Karnofsky 90–100% | 16 (24) | 52 (76) |
| 70–80% | 17 25) | 50 (75) |
| 50–60% | 6 (27) | 16 (73) |
| Sex | | |
| Male | 19 (23) | 65 (77) |
| Female | 20 (27) | 53 (73) |
| Non-radical resection | | |
| No | 34 (24) | 108 (76) |
| Yes | 5 (33) | 10 (66) |
| Age | | |
| <57 years | 12 (17) | 59 (83) |
| ⩾57 years | 27 (31) | 59 (69) |
| Weight loss | | |
| 0–5% | 28 (27) | 77 (73) |
| ⩾5% | 9 (20) | 37 (80) |
| Pulmonary symptoms | | |
| No | 8 (28) | 21 (72) |
| Yes | 31 (24) | 96 (76) |
| Extrapulmonary symptoms | | |
| No | 22 (26) | 62 (74) |
| Yes | 17 (24) | 55 (76) |

[a]VDS, vindesine; CTX, cyclophosphamide; CCNU, lomustine; MTX, methotrexate.

**Table II** Pretreatment disease parameters and disease spread evaluated for prediction of response status among 157 patients alive at 16 weeks

| Variables | No. of patients alive at 16 weeks (%) Response (n=39) | No response (n=118) |
|---|---|---|
| Disease parameters[a] | | |
| Evaluable | 11 (15) | 63 (85) |
| Measurable | 28 (34) | 55 (66) |
| Chest X-ray | | |
| No complications | 25 (26) | 70 (74) |
| Complications | 14 (23) | 46 (77) |
| Lymph node metastases | | |
| No | 11 (20) | 45 (80) |
| Yes | 27 (27) | 72 (73) |
| Brain metastases | | |
| No | 38 (27) | 104 (73) |
| Yes | 1 (7) | 14 (93) |
| Liver metastases (evidenced by ultrasonic or scintigrafic scans) | | |
| No | 39 (25) | 115 (75) |
| Yes | 0 (0) | 3 (100) |
| Bone metastases (evidenced by scintigrafic scans) | | |
| No | 35 (26) | 101 (74) |
| Yes | 4 (19) | 17 (81) |
| Bone marrow examination from posterior iliac crist | | |
| Negative | 20 (19) | 84 (81) |
| Positive | 3 (30) | 7 (70) |
| Disease extent[b] | | |
| Limited | 23 (29) | 56 (71) |
| Extensive | 16 (21) | 62 (79) |
| Metastatic sites above diaphragma | | |
| 0–1 | 23 (22) | 81 (78) |
| >1 | 16 (30) | 37 (70) |
| Metastatic sites below diaphragma | | |
| 0 | 34 (24) | 105 (76) |
| >0 | 5 (28) | 13 (72) |

[a]Measurable disease = bidimensionally measurable; evaluable disease = unidimensionally measurable. [b]Limited disease = confined to lung and regional lymph nodes including ipsilateral and contrelateral supraclavicular nodes; extensive disease = spread beyond these sites.

### *Multivariate regression analyses*

Twenty-seven pretreatment variables (Tables I–III) were chosen for analysis either because previous studies had indicated a possible effect on prognosis or because such an effect seemed likely. For each variable, the division was chosen before the analysis without knowledge of the number of responses in each groups. Thus, with respect to response prediction, the level of significance was not influenced artificially by these choices.

An analysis was performed, in which these variables were tested for prediction of response among the patients surviving 16 weeks using multiple logistic regression (Cox, 1970). Similarly, the pretreatment variables were included together with response status in an analysis of future survival among patients alive at 16 weeks using Cox's proportional hazards model (Cox, 1972). This particular group of patients was chosen for analysis in advance because a substantial part of the responses had occurred at that time and the majority of patients were still alive (Table IV).

The proportional-hazard assumption, which is a condition for applying the Cox multivariate analysis on these data, was checked by graphical and numerical methods (Anderson, 1982) and found not to be violated.

### Results

A total of 259 patients were entered into the trial. Response assessment was possible in 218 patients who had either measurable (110 patients) or evaluable disease (108 patients), and the following analysis is based on these patients. The disease parameters are listed in Table V. Fifty-three patients achieved a complete (9 patients) or partial response (44 patients) (Sørensen *et al.*, 1987). No attempt was made to distinguish between the prognostic implications of these two response types, but for the purpose of analysis patients were categorised as either responders (CR or PR) or non-responders. The responses to chemotherapy were in 50 patients documented by chest X-rays and in three patients exclusively by superficial lymph nodes (Table V). Median time to response (lasting a minimum of 4 weeks) was 10 weeks, while the median response duration was 15 weeks .

**Table III** Pretreatment laboratory values evaluated for prediction of response status among 157 patients alive at 16 weeks

| *Variables* | *No. of patients alive at 16 weeks (%)* Response ($n=39$) | No response ($n=118$) |
|---|---|---|
| Haemoglobin | | |
| $\geqslant$7.5 mM | 28 (21) | 103 (79) |
| <7.5 mM | 11 (42) | 15 (58) |
| Platelets | | |
| $400\times10^9\,l^{-1}$ | 22 (25) | 65 (75) |
| $>400\times10^9\,l^{-1}$ | 17 (24) | 53 (76) |
| White blood cell count | | |
| $\leqslant9\times10^9\,l^{-1}$ | 23 (28) | 58 (72) |
| $>9\times10^9\,l^{-1}$ | 16 (22) | 58 (78) |
| Prothrombin index | | |
| <0.70 | 3 (38) | 5 (62) |
| $\geqslant$0.70 | 17 (20) | 70 (80) |
| AST | | |
| $\leqslant40\,U\,l^{-1}$ | 37 (25) | 112 (75) |
| $>40\,U\,l^{-1}$ | 2 (20) | 8 (80) |
| Alkaline phosphatase | | |
| $\leqslant275\,U\,l^{-1}$ | 25 (24) | 81 (76) |
| $>275\,U\,l^{-1}$ | 14 (27) | 37 (73) |
| LDH | | |
| $\leqslant450\,U\,l^{-1}$ | 24 (26) | 68 (74) |
| $>450\,U\,l^{-1}$ | 10 (22) | 36 (78) |

Abbreviations: AST, aspartic aminotransaminase; LDH, lactate dehydrogenase.

**Figure 1** Comparison of survival among 39 responding (—) and 118 non-responding (---) patients alive at 16 weeks ($P=0.23$).

### *Prognostic factors for response (univariate $\chi^2$ test and multivariate logistic regression analysis)*

Among the pretreatment variables listed in Tables I–III, only two were associated with response in univariate analyses. Evaluable disease parameter ($P<0.01$), and haemoglobin level above $7.5\,\text{mmol}\,l^{-1}$ ($P<0.025$) predicted low response rates. All variables were further evaluated in a multivariate logistic regression analysis for independent prediction of response among patients surviving 16 weeks. Only the variable dividing disease parameter into measurable and evaluable disease was of independent prognostic significance for the attainment of response. The estimated probability for response was 0.16 among patients having evaluable disease and 0.33 among patients having measurable disease ($P=0.02$).

A minor influence on response rate was noted for brain metastases ($P=0.10$). Only one of 15 patients with brain metastases at study entry and still alive at 16 weeks achieved a response to chemotherapy. The probability of response was 0.37 in patients with measurable disease and no brain metastases, decreasing to 0.16 in patients without brain metastases and evaluable disease, and 0.086 and 0.029 for patients with brain metastases and measurable or evaluable disease, respectively. No other pretreatment variables were of influence for response.

**Table IV** Univariate landmark analysis of patients alive at 8, 12, 16 and 24 weeks according to response status

| *Time on study (weeks)* | *Total no. alive* | *Response status* Response | No response | *Survival responders vs. non-responders (Univariate analysis, P values)* | *Death hazard ratio responders to non-responders (95% confidence intervals)* |
|---|---|---|---|---|---|
| 0 | 218 | | | | |
| 8 | 189 | 10 | 179 | (0.78) | 0.91 (0.48–1.73) |
| 12 | 173 | 30 | 143 | (0.57) | 0.89 (0.60–1.33) |
| 16 | 157 | 39 | 118 | (0.23) | 0.79 (0.54–1.16) |
| 24 | 122 | 44 | 78 | (0.12) | 0.73 (0.49–1.09) |

*Prognostic impact of response (univariate landmark analysis and Cox's multivariate regression analysis)*

Table IV shows the response status for patients alive at different landmark times together with death hazard ratios and the *P*-values from univariate survival comparison. The median survivals for patients having a response at 8, 12, 16 and 24 weeks were 43 weeks, 43 weeks, 49 weeks and 48 weeks, while corresponding values for non-responders were 34 weeks, 36 weeks, 40 weeks and 49 weeks, respectively. The comparison of survival curves for responders versus nonresponders did not show significant differences at any time.

The relative frequency of responding patients to the total number of patients alive increased from 5.3% among those alive at 8 weeks to 36.1% at 24 weeks on study. The majority of responses (77%) occurred within the first 12 weeks, and all but one were noted before 20 weeks of treatment. Forty-four of these 52 responding patients (85%) were still alive at 24 weeks. There was no significant survival advantage for responding patients in any of the four landmark analyses.

The death hazard ratios for responding to non-responding patients at these landmark times were 0.91 (95% confidence intervals: 0.48–1.73), 0.89 (0.61–1.33), 0.79 (0.54–1.16), and 0.73 (0.49–1.09), respectively. Figure 1 shows the survival curves according to response status for all patients alive at 16 weeks, who formed the basis of the multivariate analyses.

A multivariate regression analysis was performed including response status among the 157 patients surviving 16 weeks together with the pretreatment variables from Tables I–III. Response status showed no impact on survival when evaluated against the pretreatment variables (regression coefficient = 0.1268; standard error = 0.2253; $P = 0.57$). The pretreatment variables describing performance status, non-radical resection, presence of liver metastases and LDH carried significant and independent information on survival in the Cox analysis among the 130 patients alive at 16 weeks for whom complete information was available on all these variables (Table VI). No other pretreatment variables showed any significant influence.

### Discussion

Together with response duration and overall survival, evaluation of response rates plays a key role in medical oncology, especially in phase II trials but also in phase III trials. The present state of chemotherapy for inoperable ACL indicates that at the most one-third of the patients included achieve a response to the regimens available today and complete responses seldom occur (Sørensen *et al.*, 1988*a*; Sørensen & Hansen, 1988). Assessment of the influence of various prognostic factors for response is therefore pertinent when evaluating response data, in the stratification of patients, and when selecting the inclusion criteria.

Univariate analysis of 27 variables for prediction of response in this study increases the possibility of statistical significance occurring by chance.. The finding of haemoglobin >7.5 mM as a predictor of low response rate ($P = 0.025$) may be a chance finding, and this variable is not among the significant predictors in multivariate analysis.

In the present study, a multiple logistic regression analysis was performed for patients alive at 16 weeks and classified according to response status. Only the pretreatment variable dividing disease parameter into measurable and evaluable disease was associated with significant and independent information. Ruckdeschel *et al.* (1985) observed a somewhat higher overall response rate for patients with non-small cell lung cancer having measurable disease as compared to non-measurable disease in a multiple logistic regression analysis ($P = 0.04$). This observation, however, was not confirmed in a later study by the same group of investigators (Ruckdeschel *et al.*, 1986). Eagan *et al.* (1979) observed no differences in response rates between patients with measurable or evaluable disease in a preceding univariate analysis.

It is conceivable that the lower response rate for patients with evaluable disease observed in the present study may not be caused by biological differences. Rather, it reflects the difficulties of quantitating response, as previously emphasised by Warr *et al.* (1984), especially in lesions not bidimensionally measurable. Obviously, response assessment is more difficult in partial remission than in complete remission. Unfortunately, the majority of responses in this study, as in other trials with chemotherapy in NSCLC, are only partial.

Other studies using multiple logistic regression analysis found weight loss larger than 10 kg during the preceding 3 months (Rapp *et al.*, 1988) or Karnofsky performance status and the presence of bone metastases (O'Connell *et al.*, 1986) to be of independent prognostic significance for the attainment of response.

One method of analysing the results of treatment for non-small cell lung cancer is to compare the survival for responders and non-responders (Aisner & Hansen, 1981). Unfortunately, such an analysis is associated with major methodological and interpretational pitfalls. The frequently used method of analysing survival by response, which involves dividing patients into two groups according to whether or not they ever achieve a response, is invalid (Anderson *et al.*, 1983). The method counts survival time before response as time at risk of death for the responding patient group, thereby underestimating the death rate for responders and overestimating the death rate for non-responders. As a result, neither the log rank test nor any other test for statistical significance provides a valid comparison of the risk of death in the two groups. Instead, there are two other valid methods for testing the hypothesis that responders have better survival than non-responders: the Mantel–Byar approach (Mantel & Byar, 1974) and the landmark method (Anderson *et al.*, 1983). The latter method is used in the present study.

A correlation between response and survival has previously been reported in clinical trials of patients with non-small cell lung cancer by both the Mantel–Byar method (Ruckdeschel *et al.*, 1985, 1986) and the landmark method (Ruckdeschel *et al.*, 1985). These two large studies included 432 and 486 NSCLC patients, respectively.

The present study evaluated the relationship between

**Table V** Location of disease parameters followed for response in 218 patients

| *Location of parameters* | *No. of patients (n = 218)* | *No. of responses (CR or PR) (n = 53)* |
|---|---|---|
| Chest X-ray alone | 174 | 37 |
| Chest X-ray + palpable lymph node | 34 | 11 |
| Chest X-ray + other locations | 6[a] | 2 |
| Palpable lymph node alone | 3 | 3 |
| Other locations alone | 1[b] | 0 |

[a]Cutaneous metastases, 5 patients; superficial sternal and cranial tumour, 1 patient; [b]cutaneous metastases, 1 patient.

**Table VI** Cox multivariate regression analysis of prognostic factors for future survival among ACL patients alive after 16 weeks on-study

| *Pretreatment variable* | *Regression coefficient* | *Standard error* | *P value* |
|---|---|---|---|
| Performance status = 50–60% (0/1)[a] | 1.2168 | 0.4378 | 0.0063 |
| Non-radical resection (0/1)[a] | −0.8687 | 0.3440 | 0.0062 |
| Liver metastases (0/1)[a] | 2.2827 | 0.7504 | 0.0089 |
| Ln(LDH) | 0.7334 | 0.2770 | 0.0140 |

[a]0 = no, 1 = yes.

response and survival solely in patients with ACL. No significant prognostic impact was observed for response status, although there was a trend towards longer survival for responding patients, especially among those alive at 24 weeks ($P = 0.12$). However, response status did not carry prognostic information in multivariate analyses when compared with the pretreatment variables ($P = 0.57$). In contrast, O'Connell *et al.* (1986) observed that response to platinum-based chemotherapy was strongly associated with survival among 352 patients with inoperable NSCLC of all three major histological types. Other significant pretreatment variables were performance status, LDH, sex and number of extrathoracic metastases. Whether the difference in prognostic impact of response status between the present study and that of O'Connell *et al.* is due to the different chemotherapy regimens employed, differences between the histological tumour types included or stochastic variation cannot be determined.

In the present study, patients with ACL responding to the drugs lived only slightly longer than non-responding patients, and response status was not a major predictor of future survival. An explanation of this observation may be that most of the responses, both in this study and in others (Sørensen *et al.*, 1988*a*; Sørensen & Hansen, 1988), were partial. Some caution must be exerted when intepretating these data. Firstly, a major disadvantage of the landmark method is that the results depend on the selection of an arbitrary landmark time, and conclusions of the analysis may therefore differ according to the landmark chosen (Anderson *et al.*, 1983). However, none of the four landmark analyses performed in this study showed any significant differences in survival between responding and non-responding patients.

Secondly, observer variation in response assessment may account for some of the differences in response rates observed in different studies (Warr *et al.*, 1984) and may also affect the significance of the correlation to survival. Efforts were taken to diminish errors in response assessment, by using two observers who strictly adhered to the WHO guidelines for response assessment (WHO, 1979). This, however, does not completely eliminate the possibility of observer variations.

In conclusion, a response to the cytotoxic drugs used in the present study was not a significant predictor of future survival among patients with inoperable ACL. Patients having measurable lesions had a greater likelihood of responding than patients with evaluable lesions, and patients with brain metastases seldomly responded. This may be of importance for the patient selection in future studies and for the interpretation of the results.

The authors would like to express their appreciation to the Department of Oncology, Herlev Hospital, Department of Medicine C, Bispebjerg Hospital, Copenhagen, and Department of Pulmonary Medicine, Renstromska Hospital, Gothenburg, where some of the patients in this study have been treated, and also to Jens Olsen, MD, Department of Pathology, Rigshospital, Copenhagen, for revision of the histological material. Supported by grants from the Danish Cancer Society.

### References

AISNER, J. & HANSEN, H.H. (1981). Commentary: current status of chemotherapy for non-small cell lung cancer. *Cancer Treat. Rep.*, **65**, 979.

ANDERSEN, P.K. (1982). Testing goodness of fit of Cox's regression and life model. *Biometrics*, **38**, 67.

ANDERSON, J.R., CAIN, K.C. & GELBER, R.D. (1983). Analysis of survival by tumor response. *J. Clin. Oncol.*, **1**, 710.

ARMITAGE, P. (1971). *Statistical Methods in Medical Research.* Blackwell: New York.

COX, D.R. (1970). *The Analysis of Binary Data.* Methuen: London.

COX, D.R. (1972). Regression models and life-tables. *J.R. Stat. Soc.*, **34**, 187.

EAGAN, R.T., FLEMMING, T.R. & SCHOONOVER, V. (1979). Evaluation of response criteria in advanced lung cancer. *Cancer*, **44**, 1125.

EAGAN, R.T., FRYTAK, S., CREAGAN, E.T., RICHARDSON, R.L., COLES, D.T. & JETT, J.R. (1986). Differing response rates and survival between squamous and non-squamous non-small cell lung cancer. *Am. J. Clin. Oncol.*, **9**, 249.

KAPLAN, E.L. & MEIER, P. (1958). Non parametric estimation from incomplete observation. *J. Am. Stat. Assoc*, **53**, 457.

MANTEL, N. & BYAR, D.P. (1974). Evaluation of response-time data involving transient states: an illustration using heart-transplant data. *J. Am. Stat. Assoc.*, **69**, 81.

O'CONNELL, J.R., KRIS, M.G., GRALLA, R.J. and 6 others (1986). Frequency and prognostic importance of pretreatment clinical characteristics in patients with advanced non-small-cell lung cancer treated with combination chemotherapy. *J. Clin. Oncol.*, **4**, 1604.

PETO, R., PIKE, M.C., ARMITAGE, P. *et al.* (1977). Design and analysis of randomized clinical trials requiring prolonged observation of each patient. *Br. J. Cancer*, **35**, 1.

RAPP, E., PATER, J.L., WILLAN, A. and 12 others (1988). Chemotherapy can prolong survival in patients with advanced non-small-cell lung cancer–report of a Canadian multicenter randomized trial. *J. Clin. Oncol.*, **6**, 633.

RUCKDESCHEL, J.C., FINKELSTEIN, D.M., ETTINGER, D.S. and 4 others (1986). A randomized trial of the four most active regimens for metastatic non-small-cell lung cancer. *J. Clin. Oncol.*, **4**, 14.

RUCKDESCHEL, J.C., FINKELSTEIN, D.M., MASON, B.A. & CREECH, R.H. (1985). Chemotherapy for metastatic non-small-cell bronchogenic carcinoma: EST 2575, generation V – a randomized comparison of four cisplatin-containing regimens. *J. Clin. Oncol.*, **3**, 72.

SØRENSEN, J.B., CLERECI, M. & HANSEN, H.H. (1988*a*). Single agent chemotherapy for advanced adenocarcinoma of the lung. A review. *Cancer Chemother. Pharmacol.*, **21**, 89.

SØRENSEN, J.B. & HANSEN, H.H. (1988). Combination chemotherapy for advanced adenocarcinoma of the lung. A review. *Cancer Chemother. Pharmacol.*, **21**, 103.

SØRENSEN, J.B., HANSEN, H.H., DOMBERNOWSKY, P. and 5 others (1987). Chemotherapy for adenocarcinoma of the lung (WHO III): a randomized study of vindesine versus lomustine, cyclophosphamide and methotrexate versus all four drugs. *J. Clin. Oncol.*, **5**, 1169.

SØRENSEN, J.B., HIRSCH, F.R. & OLSEN, J. (1988*b*). The prognostic implication of histologic subtyping of pulmonary adenocarcinoma according to the classification of the World Health Organization. An analysis of 259 consecutive patients with advanced disease. *Cancer*, **62**, 361.

WARR, D., McKINNEY, S. & TANNOCK, I. (1984). Influence of measurement error on assessment of response to anticancer chemotherapy: proposal for new criteria of tumor response. *J. Clin. Oncol.*, **2**, 1040.

WEISS, G.B., BUNCE, M. & HOKANSON, J.A. (1983). Comparing survival of responders and non-responders after treatment: a potential source of confusion in interpreting cancer clinical trials. *Controlled Clin. Trials*, **4**, 43.

WORLD HEALTH ORGANIZATION (1979). *WHO Handbook for Reporting Results of Cancer Treatment.* World Health Organization: Geneva.

WORLD HEALTH ORGANIZATION (1981). *Histologic Typing of Lung Tumours*, 2nd edn. World Health Organization: Geneva.

ZUBROD, C.G., SCHLEIDERMAN, M., FREI, S. and 16 others (1960). Cancer – appraisal of methods for the study of chemotherapy of cancer in man: thiophoramide. *J. Chronic Dis.*, **11**, 7.